# Neuropsychiatric and Associated Symptoms and Their Management in Caregivers of Alzheimer’s Disease Patients: A Systematic Review and Meta-Analysis

**DOI:** 10.7759/cureus.88795

**Published:** 2025-07-26

**Authors:** Aiman A Sanosi, Omar A Ayoub, Mohammed I Habadi, Jihad A Muglan

**Affiliations:** 1 Medicine, University of Jeddah, Jeddah, SAU; 2 Neurology, King Abdulaziz University Faculty of Medicine, Jeddah, SAU; 3 Family and Community Medicine, University of Jeddah, Jeddah, SAU; 4 Neurology Movement Disorders, Faculty of Medicine, Umm Al Qura University, Makkah, SAU

**Keywords:** alzheimer’s disease, anxiety, behavioral interventions, caregivers, caregiving, counselling, depression, mental health, psychiatric, sleep disturbance

## Abstract

Alzheimer’s disease (AD) patients’ caregivers often experience neuropsychiatric and other associated symptoms due to the high physical and emotional demands of caregiving. Therefore, this meta-analysis aimed to evaluate the prevalence of these symptoms and the impact of various interventions on the reduction of these symptoms. Different electronic databases, like PubMed, Scopus, ScienceDirect, and Google Scholar, were used for the search of literature using the Preferred Reporting Items for Systematic Reviews and Meta-analyses (PRISMA) guidelines. The data obtained was analyzed qualitatively to summarize the general characteristics. Meanwhile, quantitative data were analyzed using Jamovi [The jamovi project (2025), jamovi (Version 2.6) Computer Software] and RevMan 5.4 (The Cochrane Collaboration, 2020), and forest plots were generated. In addition, funnel plots were used to describe the publication bias, and for the certainty of evidence, Grading of Recommendations Assessment, Development, and Evaluation (GRADE) was used. Methodological quality assessment was performed using the Cochrane Risk of Bias (RoB-2.0) tool for randomized trials and for non-randomized trials, RoB for non-randomized Studies-Intervention (ROBINS-I). After screening, 31 studies were included in the analysis. Two types of neuropsychiatric symptoms, such as anxiety and depression, were identified. The pooled prevalence effect size was 0.40 (95% CI, 0.28-0.52, *I^2^*=89.88%, p<0.01) and 0.43 (95% CI, 0.28-0.52, *I^2^*=91.16%, p<0.01), indicating a moderate effect, and showed that 40% and 43% of caregivers experienced anxiety and depression, respectively during the care of AD patients. While a non-significant difference (p>0.01) was observed when different assessment tools were compared for the impact of various interventions, with the effect size of -0.10 (95% CI, -0.96 to 0.76) and substantial heterogeneity (*I^2^*=64%). No publication bias and methodological studies were found with low RoB; some concerns. However, some of the studies showed high RoB. Certainty of evidence was found to be moderate to high according to the GRADE framework. Neuropsychiatric symptoms are prevalent among the caregivers of AD, highlighting the need for personalized and standardized evidence-based interventions for the improvement of the well-being of the caregivers.

## Introduction and background

Alzheimer’s disease (AD), a progressive neurodegenerative disease often correlated with cognitive impairment and memory deficits, along with symptoms related to neuropsychiatry and decline in daily life functioning [1,2]. The prevalence and incidence of AD also increased from 2.92 million cases in 1990 to 7.24 million cases in 2019, and this trend was more serious in areas with a high sociodemographic index, particularly in the elderly male and female population with heart disease, diabetes, and hypertension [3]. Moreover, neuropsychiatric symptoms, including anxiety, depression, apathy, and aggression, can affect up to 90% of AD patients [4]. Since there is no proper treatment to cure AD, patients were unable to perform their routine activities and physical functions [5]. Due to these conditions, patients’ symptoms are worse, and they depend on others, even for basic life necessities [6]. As a result, the caregivers have to be engaged and more involved in maintaining the patient’s lifestyle. Notably, care for AD has also been associated with a negative impact on caregivers’ health, particularly their mental, physical, and social health [7]. In addition, spending an additional hour with AD patients increases the level of stress, depression, and anxiety [8]. Sleep disturbance symptoms like nocturnal wandering, nocturnal awakenings, and snoring also increase in caregivers and significantly impact their overall quality of life [9]. Furthermore, caregivers also developed hypertension at a much higher rate than non-caregivers [10]. Subsequently, this not only increases the workload and burnout among the caregivers, but it also has a significant impact on the quality of care provided. Therefore, preventive measures or interventions should be adopted before the symptoms become more serious.

Among interventions, the pharmacological approach plays a pivotal role, particularly in the early stages when cognitive decline may be more amenable to treatment. For instance, a moderate affinity, strong voltage-dependent memantine (N-methyl-D-aspartate receptor antagonist), which is the most commonly used drug for the treatment of AD. Likewise, intake of omega-3 fatty acids can decrease the chances of the development of AD and help manage cognitive decline, particularly in the settings of greater stress exposure [11,12]. Moreover, learning and memory impairment can be improved by the introduction of vitamin D and B12 [13]. Similarly, cholinesterase inhibitors like rivastigmine, galantamine, tacrine, and donepezil are considered cornerstones of AD treatment as well as non-AD patients [14,15]. The role of complementary therapies like Ginkgo biloba, which contain bioactive compounds and are associated with antioxidant, anti-inflammatory, and neuroprotective properties, has also proved effective for cognitive health [16].

Caregivers have a significant role in taking care of AD patients, and due to the neuropsychiatric issues they face due to care of AD patients. This not only affects the well-being of the caregivers themselves but quality of care provided to the vulnerable AD population. In addition, due to the cognitive impairment, neuropsychiatric symptoms, and other behavioral deficits in AD patients, caregivers can also be negatively impacted by caring for AD patients. They can also develop symptoms like stress, depression, anxiety, and sleep disturbance, which ultimately affect caregivers’ health, and they are also unable to provide quality care to AD patients. Therefore, early interventions are required to stabilize the caregiver’s health. Even studies are performed for the evaluation of different interventions [17-20]. However, there is a need to provide a comprehensive review of the impact of these interventions and a clear picture for physicians and policy decision-makers. This meta-analysis review aims to investigate the neuropsychiatric and associated symptoms and management in caregivers of AD patients. Consequently, this review has the potential objectives, such as assessment of the extent of neuropsychiatric and associated symptoms among caregivers of AD patients, assessment of the other behavioral disturbances among caregivers of AD patients, and assessment of the impact of different interventions among caregivers of AD patients.

In addition, the following research questions were formulated to consider the above objectives. What are the neuropsychiatric and other associated symptoms among caregivers of AD patients? What type of interventions are used, and their impact on clinical outcomes among caregivers of AD patients?

## Review

Materials and Methods

This review was performed in accordance with 27 items Preferred Reporting Items for Systematic Reviews and Meta-analyses (PRISMA) for the improvement of the quality of the review and reporting transparency [21].

Search Strategy

Different electronic databases, including PubMed, Scopus, The Cochrane Library, ScienceDirect, and Google Scholar, were searched (till January 2025) by using specific keywords and MeSH Terms like Alzheimer’s disease, neuropsychiatric symptoms (stress, depression, anxiety, sleep disturbance, sleep disorder, obstructive sleep apnea, cognitive impairment, hypertension, hypothyroidism, and delusion), intervention, management, therapy, anti-psychotic drugs, memantine, omega 3, omega three, vitamin D, vitamin B12, cholinesterase inhibitors, ginkgo biloba, and caregivers. The detailed search strategy is mentioned in Appendix 1.

Eligibility Criteria

Studies were searched according to population, exposure, and outcome (PEO) for the rate of neuropsychiatric symptoms. In this study, P represented the caregivers of AD patients, E included caring of AD patients and exposure to stressful environment, and O included development of neuropsychiatric and associated symptoms. Likewise, for management of caregiver neuropsychiatric symptoms, patient/problem, intervention, comparison, and outcome (PICO) guidelines were followed. In our study, P: included caregivers of AD patients, I: included different types of intervention used for the management of neuropsychiatric and associated symptoms among caregivers, C: control or comparator without intervention or placebo, and O: improvement in the clinical outcomes among caregivers of AD patients.

Furthermore, certain eligibility criteria were also set for the selection of studies. For instance, studies provided data associated with neuropsychiatric symptoms among caregivers. Studies provided data regarding interventions used for the management of neuropsychiatric and associated symptoms among caregivers. Randomized and non-randomized, including retrospective, observational, and cross-sectional studies in English, were included.

Similarly, certain exclusion criteria were also set for the selection of studies. For instance, studies with incomplete data or that did not include caregivers. Studies without the inclusion of AD patients or patients with other diseases, like cancer. Reviews, case studies, editorials, commentary, letters to the editor, and studies published in non-English were excluded.

Selection of Studies

**Figure 1:**
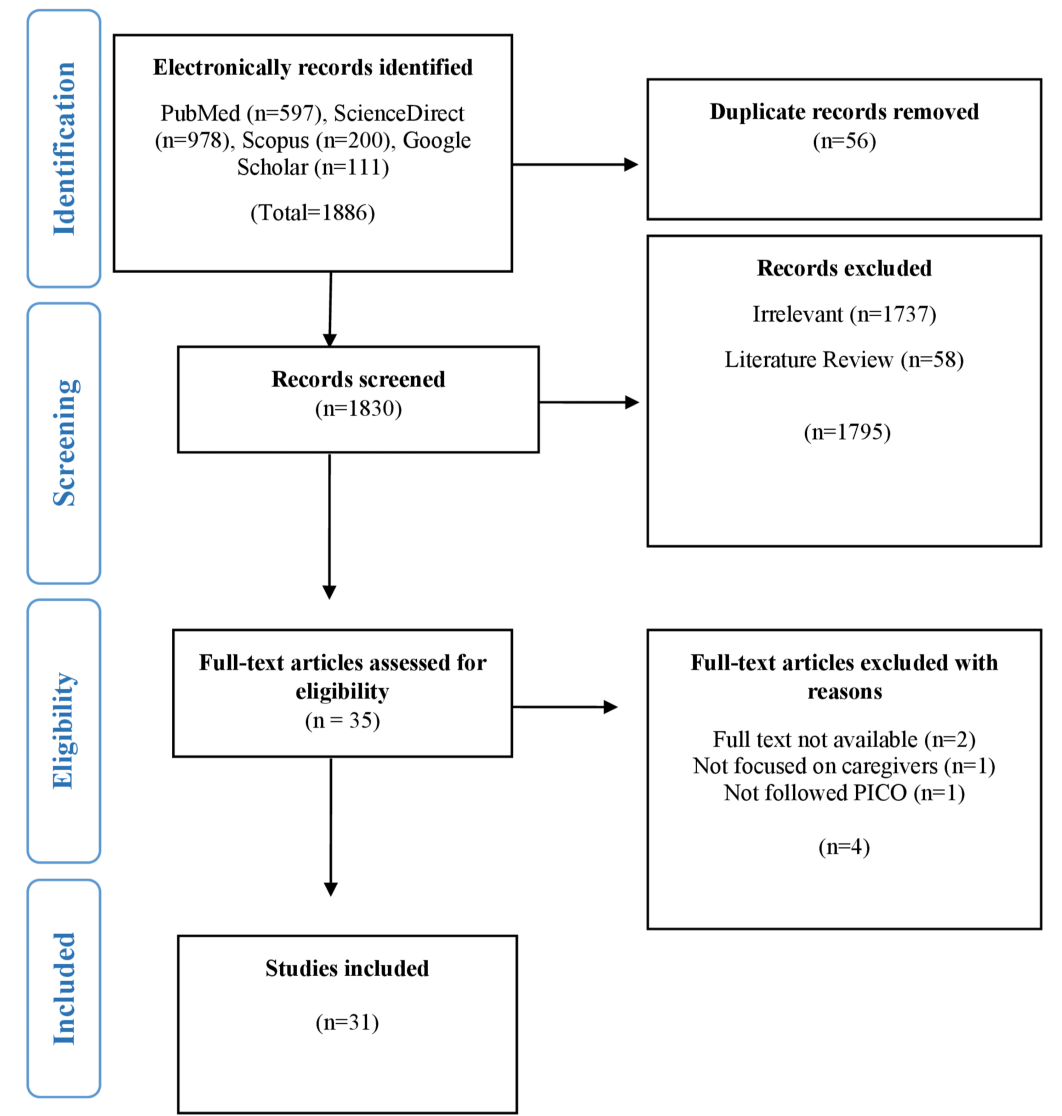
PRISMA flow chart for the selection of studies.

Studies were selected by following the PRISMA flow chart, which contains four stages for the selection of studies. In the first stage (identification), 1,886 studies were identified from different electronic databases and imported into EndNote X9 referencing software. 56 duplicate studies were identified and excluded. In the second stage (screening), 1,830 studies were screened via titles and abstracts. Irrelevant 1795 studies were excluded for reasons. The remaining 35 studies were moved to the third stage (eligibility). In this stage, the full-text assessment was made and strictly followed the eligibility criteria. Four studies were excluded and explained with reasons, and the remaining 31 studies were included for further qualitative and quantitative analysis (Figure 1). The whole process of the selection of studies was performed by two independent reviewers, and in case of any problem, a third reviewer was consulted to resolve it through discussion.

Data Extraction

Two independent reviewers used a predefined data extraction sheet, which includes characteristics of studies, participants (AD patients and caregivers), characteristics of neuropsychiatric and associated symptoms, interventions, and outcomes.

Methodological Quality Assessment

The Cochrane risk of bias-2.0 tool was used for the quality assessment of the randomized trials. Studies were assessed based on different domains and categorized as low, high, or some concerns [22]. For non-randomized studies, Risk of Bias In Non-randomized Studies - Interventions and Exposure (ROBINS-I and ROBINS-E) were used, and responses were categorized as low, high, and some concerns [23]. Further, for visualization of the assessment outcomes, robvis, a web-based tool, was used [24].

Meta-Analyses

Qualitative data were synthesized in table form, while the quantitative data were analyzed using RevMan 5.4 (Cochrane, London, UK) and Jamovi. Continuous data with the same scale for the outcomes for prevalence (number of events), intervention, and control/comparator (sample size, mean, and SD) were used for the construction of forest plots. Sub-group analyses were performed in case of different types of assessment tools used for the evaluation of interventions. Meanwhile, forest plots were constructed to display the overall effect size using the random effect model. In addition, heterogeneity was assessed by using I^2^ statistics, tau-squared (T^2^), and *X^2^* Cochrane Q tests. The I^2^ statistics were interpreted as low heterogeneity (0-29%), Moderate heterogeneity (30-49%), substantial heterogeneity (50-74%), and very high heterogeneity (75-100%). Likewise, a value of T^2^ > 1 indicated inter-study variability, and the Q test was used for the measurement of variation around the weighted means. All the analyses were performed using a random effect model and considered significant at a p-value of 0.01 [25,26]. 

Certainty of Evidence

For certainty of evidence, Grading of Recommendations Assessment, Development, and Evaluation (GRADE) was used, and outcomes of the review were categorized as high, moderate, low, and very low. Factors that can affect certainty, such as effect size, inconsistency, indirectness, and publication bias, were considered in the rating of evidence [27].

Results

General Characteristics (Prevalence of Neuropsychiatric Disorders)

Seven studies were reported from the United States of America (USA) [28-34], four studies from the United Kingdom (UK) [35-38], three studies from Brazil [39-41], three studies from Italy [42-44], two studies from Spain [45,46], each study from Canada [47], Croatia [48], Dominican Republic [49], Turkey [50], and one combine study from Australia and UK [51]. The majority of the studies followed a retrospective study design [29,33,41-44,47,48], followed by RCTs [28,30,32,38,51]. Five studies followed a cross-sectional study design [31,40,45,46,49], and the remaining studies followed different study designs as described in Table 1. A varied sample size was used in the included studies, with 30 minimum sample size and 1048 maximum sample size [34,47]. Caregivers were >50 years and belonged to the family members (spouse, son, daughter, siblings) of the patients living with the patients and spending a varied number of hours per day or week as indicated in Table 1.

**Table 1:** Summary of general characteristics of studies and participants (psychiatric symptoms). M: Male, F: Female, USA: United States of America, NA: Not Available, UK: United Kingdom, RCT: Randomized Controlled Trial.

Study ID	Country	Study design	Sample size	Age	Gender (M:F)	Education	Living not/with patients	Time spent with patients (hours)	Caregivers
Shields [34]	USA	Exploratory study	30	60.6	9:21	School and college	With patients	NA	Spouse, daughter, sister, and child
Mittelman et al. [31]	USA	Cross-sectional	206	60	86:120	NA	With patient	NA	Spouse
Kaplan and Boss [29]	USA	Retrospective	84	Males: 75.5, Females: 78.71	34:50	High school degree	No	NA	Spouse
Clyburn et al. [47]	Canada	Retrospective	1048	58.8	71% females	NA	349 with patient in community and 699 in institute	NA	Family members and informed caregivers
Marriott et al. [38]	UK	RCT	28 (Intervention group=9, Control 1=11, Control 2=9)	28 (Intervention group=69.6, Control 1=58.1, Control 2=63)	0:28	NA	NA	NA	Spouse, offspring, siblings
Powers et al. [33]	USA	Retrospective	51	63.39	14:37	14.02 years schooling	With patient	NA	Spouse
Eisdorfer et al. [28]	USA	RCT	225	69	25%:75%	49% had high school education	With patient	NA	Spouse, offspring, siblings
Mahoney et al. [30]	USA	RCT	100 (Intervention group: 49, Control group: 51)	Intervention group: 61.4, Control group: 63.7	Intervention group: 9:40, Control group: 13:38	High school	With patient	NA	Spouse, siblings, child
Aguglia et al. [42]	Italy	Retrospective	236	Males: 64.7, Females: 61.1	77:158	Males: 10.9 years for schooling, Females: 10.3 years	With patients	NA	Spouse, daughter-in-law, grandchild, sister, and child
Mittelman et al. [32]	USA	RCT	406	71.3	162:244	NA	With patients	NA	Spouse
Mahoney et al. [37]	UK	Naturalistic study	153	64	46:107	NA	With patients	NA	Spouse, daughter, sister, son, and friends
Cooper et al. [35]	UK	Comparative	126 (Anxiety group: 40, No anxiety group: 86)	>65	82:44	NA	The majority are living with patients	Anxiety group: 16.4, No anxiety group: 13.1	Spouse, offspring, siblings
Cooper et al. [36]	UK	Longitudinal study	126 (Re-interviewed:93, Not re-interviewed:33)	Re-interviewed:63.9, Not re-interviewed:65	Re-interviewed:34:59, Not re-interviewed:10:23	NA	With patient	NA	Spouse
Brodaty et al. [51]	Australia and UK	RCT	155 (intervention group=79, Control group=76)	Intervention group=71.5, Control group=71.1	Intervention group=33:46, Control group=36:40	NA	No	NA	Spouse
García-Alberca et al. [46]	Spain	Cross-sectional	125	61.41	26:99	NA	With patients	NA	Spouse, husband, son, daughter
García-Alberca et al. [45]	Spain	Cross-sectional	80	62.15	18:62	6.56 years schooling	NA	12.70	Spouse, husband, son, daughter, brother, sister
Ostojić et al. [48]	Croatia	Retrospective	30	57.6	8:22	High school and university	63.3% lived with patients	16.43	Child and spouse
Medrano et al. [49]	Dominican Republic	Cross-sectional	67	61	11:56	Elementary education and professional caregivers	NA	13-16	Family (spouse, son, daughter, sibling, grandchild)
Iavarone et al. [43]	Italy	Retrospective	86	57.5	37:49	Schooling for 12 years	NA	NA	Spouse and offspring
Raggi et al. [44]	Italy	Retrospective	73	64	28:45	Schooling for 8 years	100% living with patients	NA	Family (spouse, son, daughter, sibling, nephew)
Corrêa et al. [39]	Brazil	Comparative	Caregivers=17, Non-caregivers=18	Caregivers=64.83, Non-caregivers=58.29	Caregivers=5:13, Non-caregivers=3:14	NA	NA	125 hours/weekly	Family members
Bozgeyik G et al. [50]	Turkey	Prospective and clinic-based study	71	52	13:58	Schooling for 8 years	With patient	NA	Daughters, spouse, son, daughter-in-law
Delfino et al. [41]	Brazil	Retrospective	134	58.24	27:107	Schooling for 14 years	58% of caregivers live with patients	Most spend >16	Daughters, spouse, husband, son
de Araujo and Lacerda [40]	Brazil	Cross-sectional	49	54.26	0:49	Schooling for > 8 years	with patient	Between 16-24	Daughters and sole caregivers

General Characteristics (Management Strategies)

Six studies were reported from the USA [28,30,31,52-54], followed by three studies from the UK [35,36,38], two studies from Switzerland [55,56], a single study from Hong Kong, France [57,58], and a combined study from Australia and the UK [51]. The majority of the studies followed a randomized study design [28,30,38,51,53-58], and others followed study designs that are described in Table 2.

**Table 2:** Summary of general characteristics of studies and participants (management strategies). M: Male, F: Female, USA: United States of America, NA: Not Available, RCT: Randomized Controlled Trial, UK: United Kingdom.

Study ID	Country	Study design	Sample size	Age	Gender (M:F)	Education	Living not/with patients	Time spent with patients	Caregivers
Mittelman et al. [31]	USA	Cross-sectional	206 (Interventional group=103, Control group=103)	60	86:120	NA	With patient	NA	Spouse
Marriott et al. [38]	UK	RCT	28 (Intervention group=9, Control 1=11, Control 2=9)	28 (Intervention group=69.6, Control 1=58.1, Control 2=63)	0:28	NA	NA	NA	Spouse, offspring’s, siblings
Shikiar et al. [58]	France	RCT	603 (Intervention 60/80 mg=189, 40/50 mg=197, Control=205)	Intervention 60/80 mg=64.1, 40/50 mg=62.7, Control=63.3	Intervention 60/80 mg=87:102, 40/50 mg=75:122, Control=87:118	NA	NA	NA	Spouse, siblings
Eisdorfer et al. [28]	USA	RCT	225	69	25%:75%	49% had high school education	With patient	NA	Spouse, offspring’s, siblings
Mahoney et al. [30]	USA	RCT	100 (Intervention group: 49, Control group: 51)	Intervention group: 61.4, Control group: 63.7	Intervention group: 9:40, Control group: 13:38	High school	With patient	NA	Spouse, siblings, child
Mittelman et al. [53]	USA	RCT	406 (Intervention group=203, Control group=203)	Intervention group=71.5, Control group=71.1	Intervention group=92:111, Control group=70:133	A majority completed high school	NA	NA	Spouses
Akkerman and Ostwald [52]	USA	Comparative	35 (Intervention group=18, Control group=17)	58.1	5:.33	14.14 years of education	NA	111 hours weekly	Spouse
Cooper et al. [35]	UK	Comparative	126 (Anxiety group: 40, No anxiety group: 86)	>65	82:.44	NA	Majority living with patients	Anxiety group: 16.4, No anxiety group: 13.1	Spouse, offspring’s, siblings
Cooper et al. [36]	UK	Longitudinal study	126 (Re-interviewed:93, Not re-interviewed:33)	126 (Re-interviewed:63.9, Not re-interviewed:65)	126 (Re-interviewed:34:59, Not re-interviewed:10:23)	NA	With patient	NA	Spouse
Brodaty et al. [51]	Australia and UK	RCT	155 (intervention group=79, Control group=76)	Intervention group=71.5, Control group=71.1	Intervention group=33:46, Control group=36:40	NA	No	NA	Spouse
Williams et al. [54]	USA	RCT	116 (Intervention group=59, Control group=57)	Intervention group=62.1, Control group=59	Intervention group=44:15, Control group=46:11	High school to post-graduate	Majority living with patients	NA	Spouse and child
Aboulafia-Brakha et al. [55]	Switzerland	RCT	27 (Intervention group=12, Control=15)	Intervention group=59.42, Control group=55.07	Intervention group=0:15, Control group=	NA	NA	Intervention group=6 hours, Control group=5.40 hours	Spouse, parents
Cheng et al. [57]	Hong Kong	RCT	103 (Intervention group=34, Control 1=36, Control 2=33)	56.16	86% females	Primary to tertiary	NA	70.19 hours per week	Spouse, children and children-in-laws
Forstmeier et al. [56]	Switzerland	RCT	41 (Intervention=20, Control=21)	Intervention=74.90, Control=76.19	Intervention=7:13, Control=8:13	12.72 years of schooling	Partial	NA	Spouse, children

Furthermore, the varied sample sizes were used in the included studies, with 603 maximum of caregivers randomized into three groups (two dose regimes and control group) [58], and 27 were minimum caregivers randomized into two groups (intervention and control groups) [55]. Female caregivers who belonged to the family (spouse, child, siblings) were the dominant gender with high school education, living with patients, spending different time durations as indicated in Table 2.

Characteristics of Neurological Symptoms and Outcomes

Table 3 summarizes the characteristics of neuropsychiatric and other associated symptoms experienced by caregivers, along with tools used for the assessment of the level of these neuropsychiatric symptoms and the outcomes. Depression was found to be the most prevalent neuropsychiatric condition among caregivers [28,29,31-34,47,50,51], followed by anxiety [35,36,43]. Meanwhile, caregivers also showed symptoms of anxiety, depression, stress, and distress, as described in Table 3.

**Table 3:** Summary of characteristics of neuropsychiatric and associated symptoms and prevalence. NA: Not Available, CESD: The Center for Epidemiological Studies for Depression, BDI: Beck Depression Inventory, STAI: State-Trait Anxiety Inventory, CBI: The Caregiver Burden Inventory, BSI: The Brief Symptom Inventory, HADS: Hospital Anxiety and Depression Scale, HADS-A: Hospital Anxiety and Depression Scale - Anxiety, HADS-D: Hospital Anxiety and Depression Scale-Depression, HDRS: Hamilton Depression Rating Scale, BAI: Beck Anxiety Inventory, NPI: Neuropsychiatric Inventory, HAM-D: Hamilton Depression Rating Scale, DASS-21: Depression, Anxiety and Stress Scale.

Study ID	Neurological symptoms type	Associated symptoms or other diseases	Scale used for severity of neuro-symptoms	Scores (Mean±SD)	Conclusion
Shields [34]	Depression	NA	CESD	CESD=34.4±9.9	Attitude of the family members (angry and sad responses) accounted for over 44% of increase in caregiver depressive symptoms
Mittelman et al. [31]	Depression	Physical health	Symptoms	9.75±6.47	Caregivers had depression
Kaplan and Boss [29]	Depression	NA	CESD	CESD=13.76±10.06	Caregivers were found with depression
Clyburn et al. [47]	Depression	Disturbing behavior	The Burden Interview, CESD	The Burden Interview=15.15±13.33, CESD=6.91±8.16	Patient’s caregivers showed more disturbing behaviors and functional limitations working within community while, those working an institution had more informal support.
Marriott et al. [38]	Distress and depression	Burden	BDI	11.5±9.5	High level of distress and depression among caregivers
Powers et al. [33]	Depression	NA	BDI	6.02±5.32	Caregivers have depression
Eisdorfer et al. [28]	Depression	NA	CESD	Cuban American: 17.83±8.9, White American: 17.60±11.9	High levels of clinically significant depressive symptoms
Mahoney et al. [30]	Anxiety and depression	NA	CESD and STAI	CESD=13.7±11.1, STAI=20.9±6.8	High level of depression and anxiety among caregivers
Aguglia et al. [42]	Stress, depression, distress	NA	CBI, BSI	Male: CBI=23.8±4.3, Anxiety (BSI)=5.1±1.5, Depression (BSI)=5±1.3; Female: CBI=26.9±5.1, Anxiety (BSI)=6.1±1.2, Depression (BSI)=5.3±0.9	Increased level of the caregiver’s distress, anxiety, and depression
Mittelman et al. [32]	Depression	NA	Geriatric Depression Scale	9.8±6.6	Caregivers had depression
Mahoney et al. [37]	Anxiety and depression	NA	HADS	HADS-A=7.4±4.4, HADS-D=5.1±4	Caregivers, particularly females, had high levels of anxiety and depression
Cooper et al. [35]	Anxiety	NA	HADS-A	NA	Caregivers have anxiety
Cooper et al. [36]	Anxiety	Burden	HADS-A	Re-interviewed: 5.7±4.0, Not re-interviewed: 7.8±5.5	No significant difference between groups
Brodaty et al. [51]	Depression	NA	BDI	Intervention group: 9.2±7.02, Control group: 8.3±6.05, Total: 8.7±6.53	Caregivers have depression
García-Alberca et al. [46]	Anxiety and depression	Burden	HDRS, STAI	HDRS=17.12±6.76, STAI=35.59±7.05	Caregivers have shown high depression and anxiety scores
García-Alberca et al. [45]	Anxiety and depression	Burden	BDI and STAI	BDI:28.11±8.76, STAI: 34.50±11.61	Higher anxiety and depression scores among caregivers
Ostojić et al. [48]	Anxiety and depression	NA	HADS	HADS-A=10.46±4.26, HADS-D=8.03±4.08	Caregivers had anxiety and depression symptoms and should also be provided due care and support.
Medrano et al. [49]	Depression, anxiety	Burden	HARS	HARS-D=44%, HARS-A=19%	Caregiver burden was associated with anxiety and depression
Iavarone et al. [43]	Anxiety	Burden	CBI, STAI	NA	AD is associated with high anxiety among caregivers
Raggi et al. [44]	Distress	CVD, Endocrinologic, Rheumatic, Neoplastic, Gastrointestinal, Pneumologic	CBI and the Neuropsychiatric Inventory Caregiver Distress Scale	CBI=33, Neuropsychiatric Inventory Caregiver Distress Scale=4	Patient with high behavioral disturbance are associated with increasing burden and distress levels in their caregivers
Corrêa et al. [39]	Stress, depression, anxiety	Exhaustion and physical	BDI, BAI	Caregivers: BDI=15.88±1.27, BAI=9.00±1.19; Non-caregivers: BDI=5.82±0.80, BAI=3.47±0.87	Caregivers had higher levels of stress, depression, and anxiety symptoms than non-caregivers
Bozgeyik G et al. [50]	Depression	Burden	HAM-D	HAM-D: Mild: 19.7%, Moderate: 19.7%, Severe: 4.2%	Patients' disturbing behavior increases the burden and causes caregiver depression
Delfino et al. [41]	Depression and distress	Burden	BDI, NPI	BDI: 6.26 (5.98), NPI: 13 (9.07)	Overburdened caregivers are associated with symptoms of depression and distress
de Araujo and Lacerda [40]	Depression, anxiety, stress	Burden	DASS-21	Depression=65.31%, Anxiety=53.06%. Stress=55.10%	Caregivers had presented more symptoms of depression, anxiety, and stress

Similarly, other associated symptomology included burden (stress due to work), physical health (chronic pain, digestive problems), and other disturbing behaviors, like depression, distress, and stress [31,38,39,44-47]. In addition, a varied number of assessment tools were identified for depression and anxiety, such as The Center for Epidemiological Studies for Depression (CESD), Hamilton Depression Rating Scale (HDRS), State-Trait Anxiety Inventory (STAI), Hospital Anxiety and Depression Scale (HADS), Beck Depression Inventory (BDI), Beck Anxiety Inventory (BAI), Neuropsychiatric Inventory (NPI, and Depression, Anxiety and Stress Scale (DASS-21). Using these assessment tools, varied and high values were observed among the caregivers, which indicated a high level of neuropsychiatric symptoms among caregivers (Table 3).

Characteristics of Interventions Used for the Management of Neuropsychiatric Symptoms and Outcomes

Depression was the leading neuropsychiatric symptom among caregivers [28,31,51,56,57], followed by anxiety [35,36,52]. In addition, distress, behavioral problems, and a combination of depression and anxiety were also identified among caregivers [30,38,53-55,58]. Furthermore, other associated neuropsychiatric symptoms, like anger, physical health, burden, sleep disturbance, and functional disabilities [31,38,54,56,57]. A varied number of interventions were identified, which were used for reducing neuropsychiatric symptoms among the caregivers. For instance, Cognitive Behavioral Therapy (CBT) [52,55,56], technology-based interventions [28,30,54], coping strategies [35,36], and others are explained in Table 4.

**Table 4:** Summary of characteristics of interventions used for the management of neuropsychiatric and associated symptoms. GHQ: General Health Questionnaire, NA: Not Available, aRSS: An abridged version of the Relatives Stress Scale, SET: Structural Ecosystems Therapy, CIIT: Structural Ecosystems Therapy + Computer Telephone Integrated System, RMBPC: The Revised Memory and Behavior Problems Checklist, CBT: Cognitive Behavioral Therapy, BAI: Beck Anxiety Inventory, HAMA: Hamilton Anxiety Scale, COPE: Coping Orientation to Problems Experienced, HSQ: Health Status Questionnaire, BDI: Beck Depression Inventory, CESD: The Center for Epidemiological Studies for Depression, STAI: State-Trait Anxiety Inventory, STAIS: State-Trait Anxiety Inventory – State, PSS: Perceived Stress Scale, HDRS: Hamilton Depression Rating Scale, BFT: Benefit Finding Intervention, CSDD: Clinician-rated Depression.

Study ID	Neurological symptoms type	Associated symptoms or other diseases	Management strategies	Intervention composition	Duration	Assessment tool	Outcomes	Follow-up	Conclusion
Mittelman et al. [31]	Depression	Physical health	A comprehensive support program	Counseling: highlighting caregivers' role and education, Problem solving: Trained to prevent problems associated with patient behaviors	Every four months in the first year and every 6 months	No standardized tool was used	Baseline-12-month follow-up, average change = -0.10	12 months	Change in depression level was observed in intervention group
Marriott et al. [38]	Distress and depression	Burden	Cognitive-behavioral family intervention	Three main components: caregiver’s education, coping skills training, and stress management	14 sessions with two-week intervals	GHQ	Intervention=5.1±5.5, Control=12.4±6.4	12 months	Cognitive-behavioral family intervention has a significant impact
Shikiar et al. [58]	Distress	NA	Pharmacological	Metrifonate Therapy (60/80 and 40/50 mg)	26 weeks	aRSS	Intervention 60/80 mg=9.7±6.7, 40/50 mg=10.1±7.2, Control=9.6±7	No follow-up	Significantly reduced the psychological burden of caregivers
Eisdorfer et al. [28]	Depression	NA	Family Therapy and Technology-Based Intervention	SET, CIIT	NA	RMBPC	Cuban American: Minimal support group: 2.14±2, SET-CA: 1.99±1.7, CIIT-CA: 2.45±2; White American: Minimal support group: 1.90±1, SET WA: 1.73±1.5, CIIT-WA: 1.85±1.5	18 months	Combined therapy significantly reduces depressive symptoms among caregivers
Mahoney et al. [30]	Anxiety and depression	NA	Technology intervention	Computer-mediated automated interactive voice response	1 year	RMBPC	Intervention group: 12.2±11, Control group: 12.3±13.1	18 months	No significant effect of the intervention in reducing bother RMBPC, CES-D, or STAI-A scores
Mittelman et al. [53]	Behavioral problems	NA	Counseling	Individual and family counseling, continuous emotional support and education	Every 4 months during the first year, and every 6 months thereafter	RMBPC	Intervention group: 41.2±18.3, Control group: 46.7±19.4	NA	Significant impact of intervention
Akkerman and Ostwald [52]	Anxiety	NA	CBT	Didactic skills training and used a multidimensional model to address the physical, cognitive, and behavioral components associated with caregiver anxiety through	Two-hour weekly meetings for 9 weeks	BAI and HAMA	BAI: Intervention group=7.72±5.37, Control group=14.41±9.08; HAMA: Intervention group=14.44±9.56, Control group=27.24±10.6	26 Weeks	Intervention leads to sustained benefits in reducing anxiety
Cooper et al. [35]	Anxiety	NA	Coping strategies	Emotion and problem-focused strategies	NA	Brief COPE and HSQ	Mean COPE dysfunction scores were significantly higher for caregivers	18 months	Improved anxiety symptoms after intervention
Cooper et al. [36]	Anxiety	Burden	Coping strategies	Emotion-focused strategies and more problem-focused strategies	NA	Brief COPE and HSQ	Psychological intervention package to emphasize emotion-focused coping may be a rational approach to reducing anxiety	30 months	Significant impact on reducing anxiety symptoms
Brodaty et al. [51]	Depression	NA	Counseling	NA	Five sessions within 3 months	BDI	No significant difference between intervention and control	5.4 years	No differences in nursing home placement or mortality by intervention group
Williams et al. [54]	Depression and anxiety	Anger, perceived stress, hostility, sleep disturbance	Video-Based Coping Skills	10 coping skills	NA	CESD, STAI	CESD: Interventional group=18.7±10.6, Control group=14.4±9.6; STAI: Interventional group=41.9±11.1, Control group=37.3±12.5	NA	Significant impact of intervention in reducing depression and anxiety
Aboulafia-Brakha et al. [55]	Stress	NA	CBT	Cognitive restructuring and behavior modifications	8 weekly sessions	BDI, STAIS, PSS	BDI: Intervention group=9.4±5.7, Control group=7.9±4.8, STAIS: Intervention group=45±8.6, Control group=39±9; PSS: Intervention group=19.5±5.3, Control group=16.8±7.1	12 months	CBT attenuates psychophysiological responses to stressful situations in caregivers, by reducing diurnal cortisol levels
Cheng et al. [57]	Depression	Burden, psychological well-being	BFT	NA	Four biweekly sessions	HDRS Chinese version	Not clear	12 months	Significantly reduce depressive symptoms in caregivers
Forstmeier et al. [56]	Depression	Apathy, other neuropsychiatric symptoms, functional abilities	CBT	Behavioural activation, behaviour management, interventions for the caregiver, reminiscence, couples counselling, and cognitive restructuring	25 weekly sessions	CSDD	Intervention group=6.80±4.20, Control group=7.96±7.09	12 months	Outcomes are encouraging

Additionally, varied assessment tools, such as the Revised Memory and Behavior Problems Checklist (RMBPC), General Health Questionnaire (GHQ), brief Coping Orientation to Problems Experienced (COPE) and Health Status Questionnaire (HSQ), The Center for Epidemiological Studies for Depression (CESD), and Beck Anxiety Inventory (BAI), were utilized for the neuropsychiatric symptoms and to compare the outcomes of intervention. Overall, improvement was observed in scores after intervention, and all of the studies demonstrated the impact of these interventions on the reduction of neuropsychiatric symptoms in caregivers, except for two studies, which found no significant difference between intervention and control groups [30,51] (Table 4).

Meta-Analysis (Prevalence)

**Figure 2:**
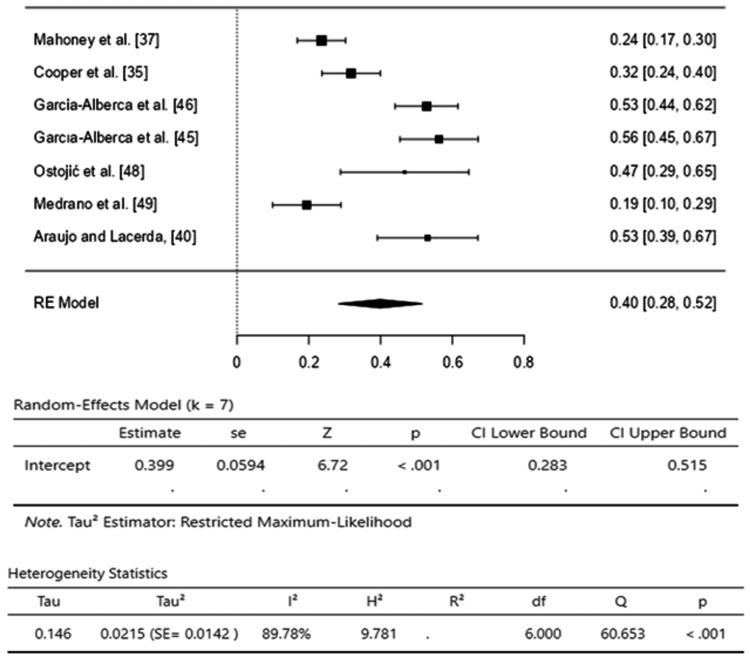
Forest plot for anxiety prevalence among caregivers of Alzheimer’s disease patients. [35,37,40,45,46,48,49]

Anxiety: The pooled effect size of seven studies [35,37,40,45,46,48,49] was 0.40 (95% CI, 0.28-0.52), indicating a moderate effect, and showed that 40% of caregivers experienced anxiety during the care of Alzheimer’s disease patients. However, high heterogeneity was observed among the studies (I2=89.88%) with a significant Q-test (p<0.01), and T2 (0.021) quantifies between-study variance (Figure 2).

**Figure 3:**
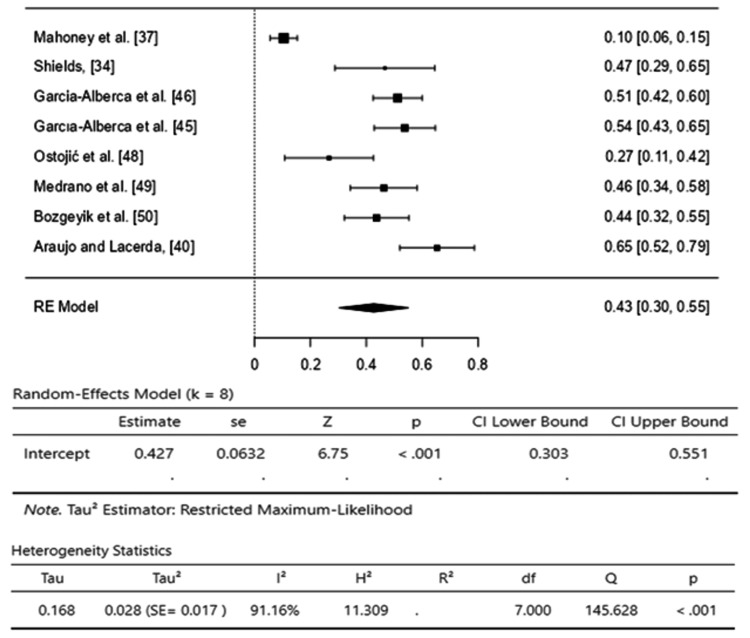
Forest plot for depression prevalence among caregivers of Alzheimer’s disease patients. [34,37,40,45,46,48-50]

Depression: The pooled effect size of eight studies [34,37,40,45,46,48-50] was 0.43 (95% CI, 0.28-0.52), indicating a moderate effect, and showed that 43% of caregivers experienced depression during the care of Alzheimer’s disease patients. However, high heterogeneity was observed among the studies (I2=91.16%) with a significant Q-test (p<0.01), and T2 (0.028) quantifies between-study variance (Figure 3).

Meta-Analysis (Assessment Tools Used for Impact Assessment of Different Interventions)

The pooled effect size for the GHQ assessment tool used for comparison of the outcomes of the intervention used for the management of neuropsychiatric symptoms in caregivers of Alzheimer’s disease patients was -6.63 (95% CI, -10.30 to -2.87) with low heterogeneity (I2=0%) and significant difference (p<0.01) [38]. Likewise, the pooled effect size of three studies [28,30,53] for RMBPC was -0.08 (95% CI, -0.55 to 0.77) with substantial heterogeneity (I2=50%), and a non-significant difference was observed (p>0.01).

**Figure 4:**
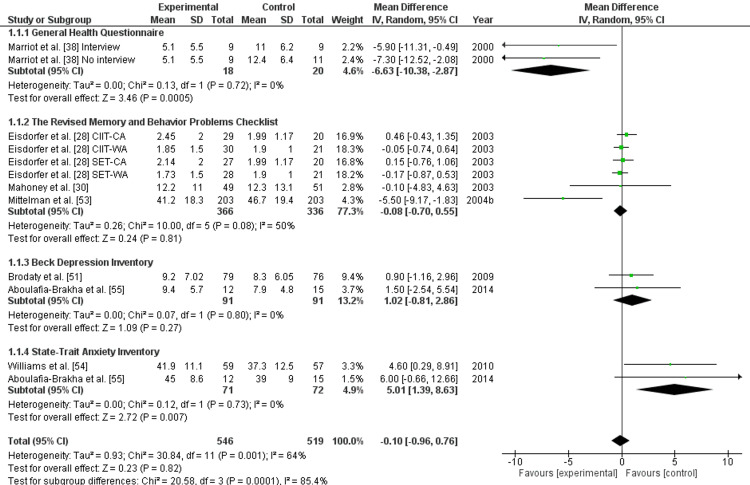
Forest plot for comparison between different assessment tools used for the evaluation of interventions for reducing neuropsychiatric symptoms in caregivers of Alzheimer’s disease patients. [28,30,38,51,53-55]

Furthermore, the pooled effect size of two studies [51,55] for BDI was 1.02 (95% CI, -0.81 to 2.86) with low heterogeneity (I2=0%) and non-significant difference (p>0.01). Moreover, the pooled size of two studies [54,55] for STAI was 5.01 (95% CI, 1.39 to 8.63) with a significant difference (p<0.01) and low heterogeneity (I2=0%). Overall, a non-significant difference (p>0.01) with an effect size of -0.10 (95% CI, -0.96 to 0.76) and substantial heterogeneity (I2=64%) as indicated in Figure 4.

Publication Bias for Anxiety and Depression Prevalence Studies

**Figure 5:**
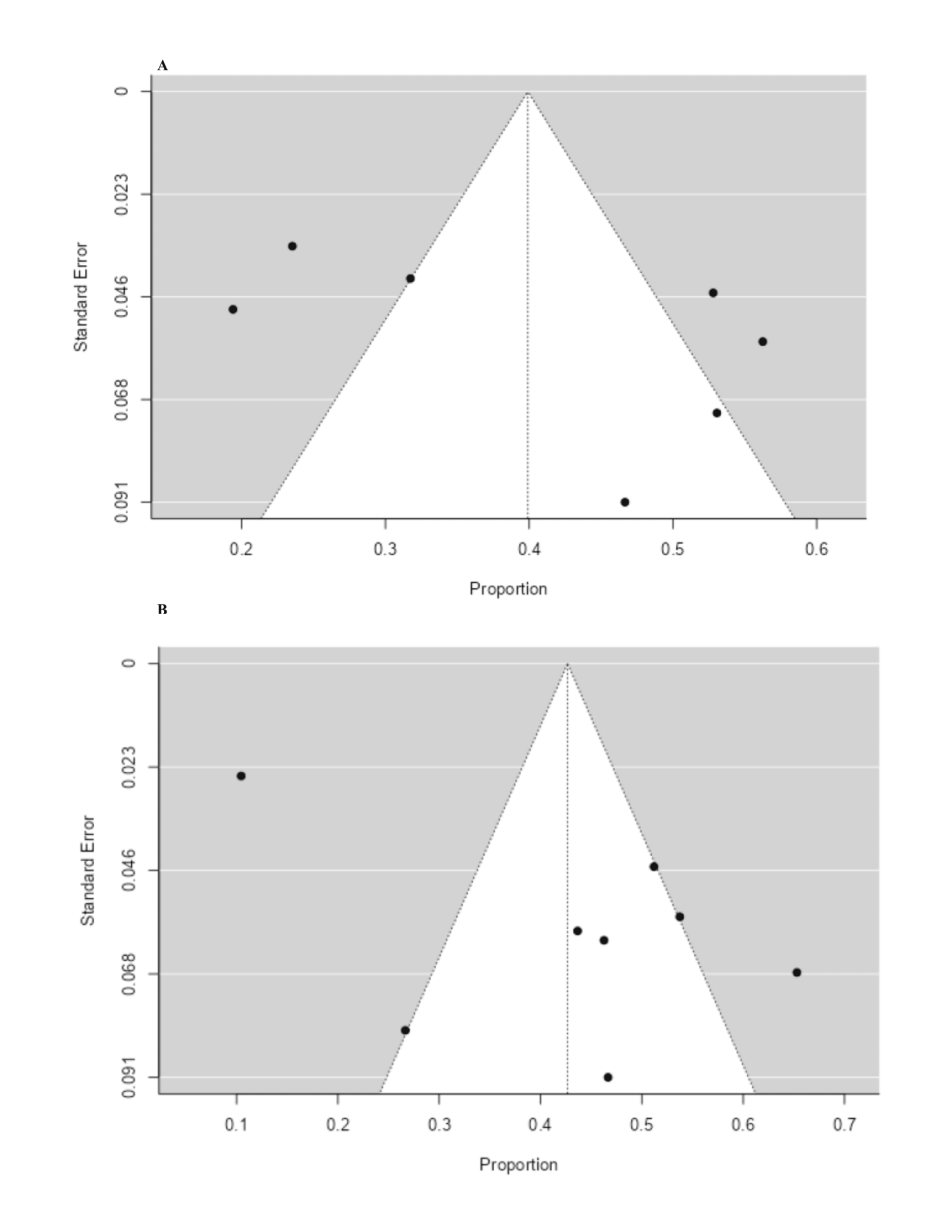
Publication bias for studies reported prevalence. A. Anxiety, B. Depression.

The rank correlation test for funnel plot asymmetry (Kendall T) for studies reporting anxiety was 0.23 (p=0.56), demonstrating no significant evidence of funnel plot asymmetry, indicating a lack of publication bias. Likewise, the regression test (Z=1.33, p=0.18) also fails to detect significant asymmetry, reinforcing the conclusion that publication bias had no impact on the meta-analysis (Figure 5A). Similarly, the rank correlation test for funnel plot asymmetry (Kendall T) for studies reporting depression was -0.07 (p=0.90), indicating non-significant asymmetry and had no impact on publication bias. Likewise, the regression test also did not show any significant evidence of publication bias (Z=1.15, p=0.25), as described in Figure 5B.

Publication Bias for Management Studies

**Figure 6:**
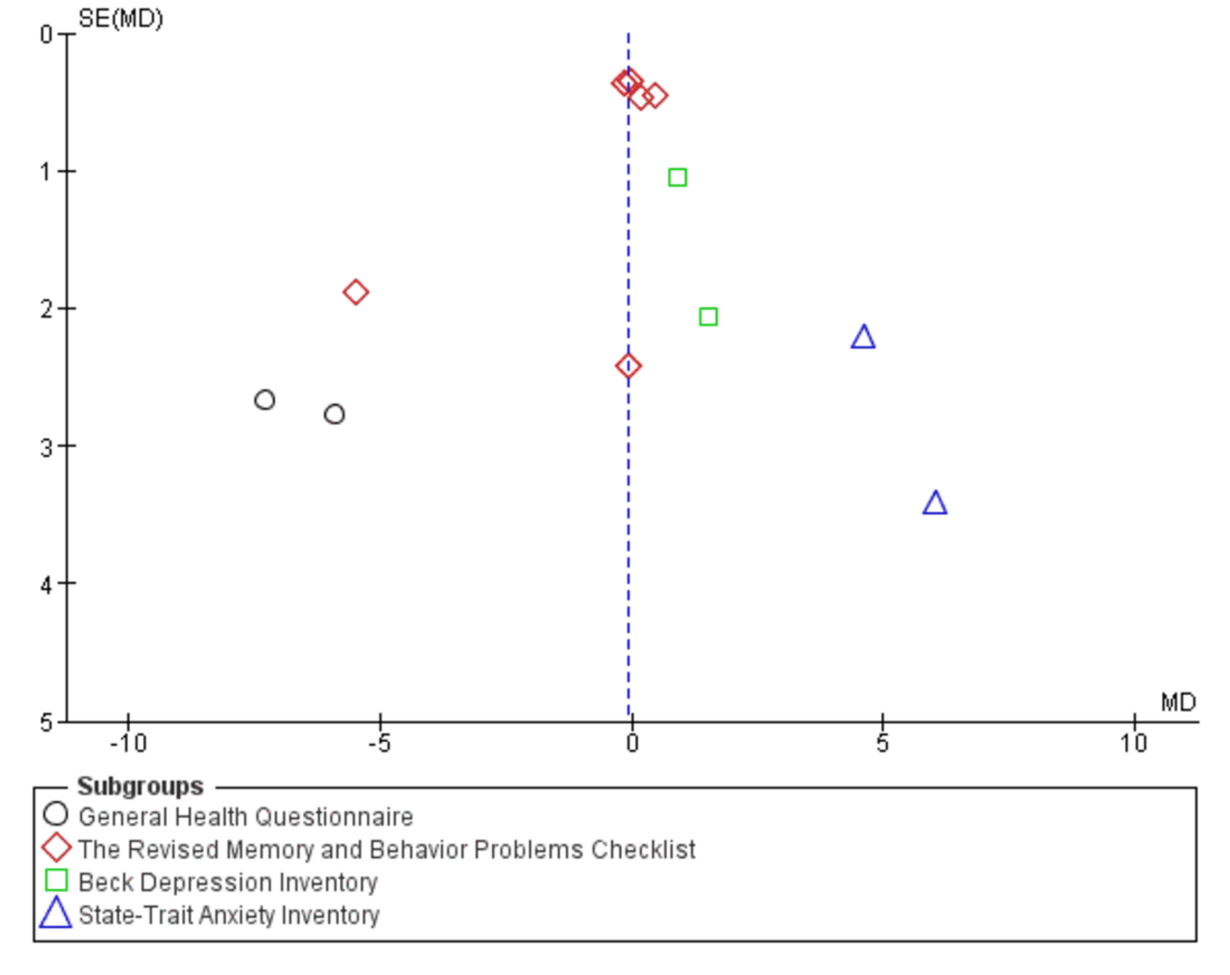
Funnel plot for studies addressing management strategies for reducing neuropsychiatric symptoms among caregivers of Alzheimer’s disease patients.

All studies were distributed around the symmetrical center line, with large and small studies balanced on either side of the effect size line, forming a clear funnel shape. This symmetrical distribution of studies around the average effect size suggests low publication bias. This raises the possibility that both positive findings and negative results were adequately represented in the meta-analysis, enhancing its overall reliability (Figure 6).

Methodological Quality Assessment

**Figure 7:**
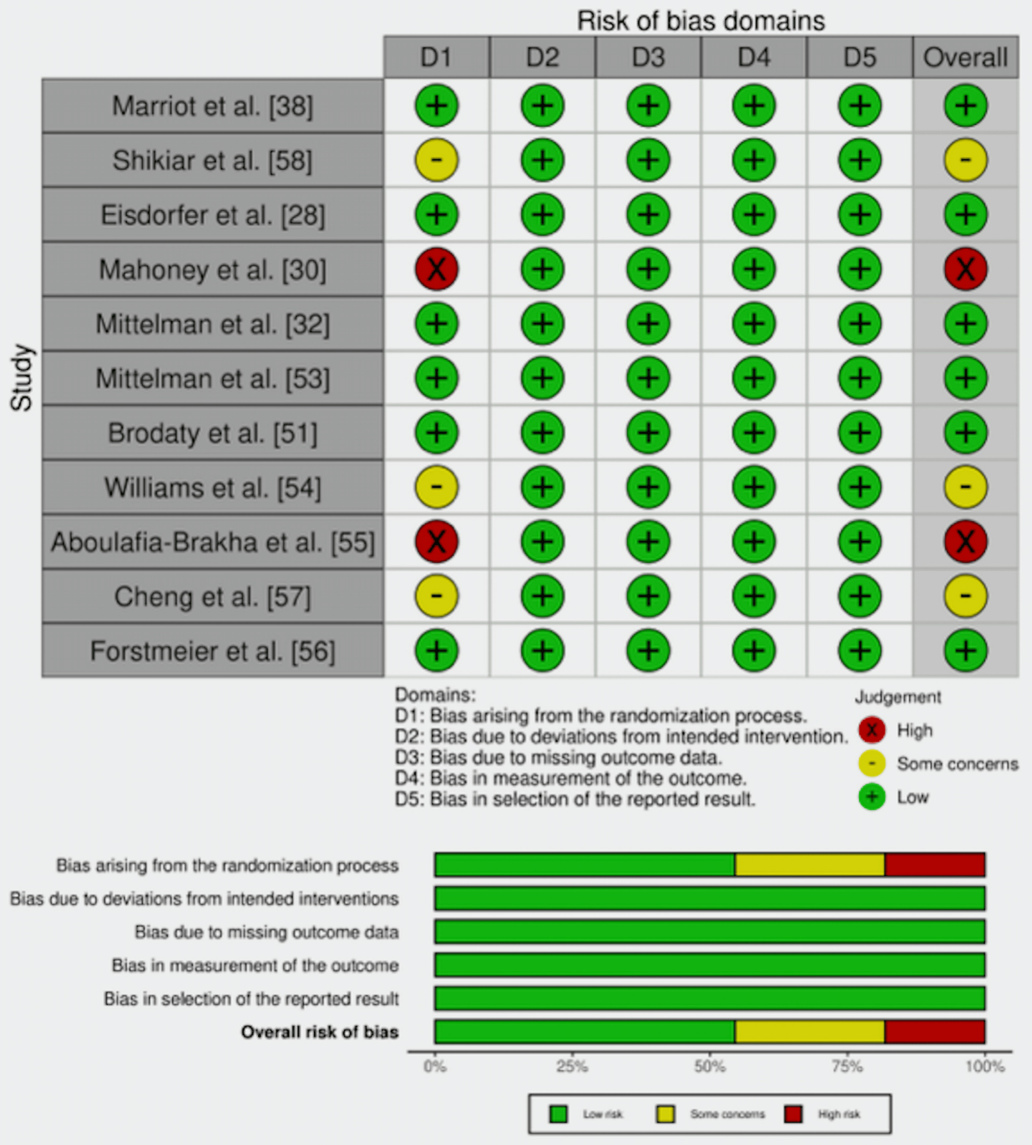
Methodological quality assessment using the Cochrane RoB-2.0 for randomized studies. [28,30,32,38,51,53-58]

Randomized studies: Most of the studies had low RoB [28,32,38,51,53,56]. Three studies had some concerns in the process of randomization, as these studies only mentioned randomization and did not explain the process used for randomization [54,57,58]. Meanwhile, two studies had high RoB in the randomization bias [30,55], as described in Figure 7.

**Figure 8:**
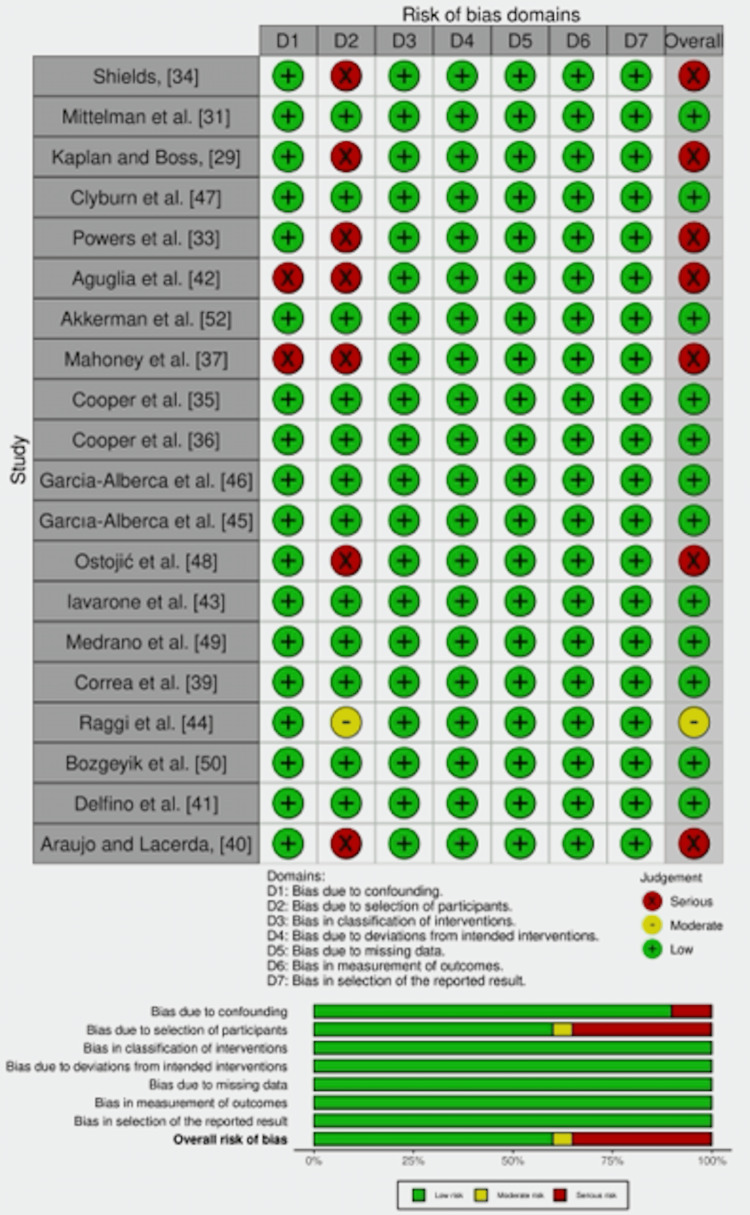
Methodological quality assessment using the ROBINS-I for non-randomized studies. [29,31,33-37,39-50,52]

Non-randomized studies: Overall, 13 studies were found with low RoB [31,35,36,39,41,43,45-47,49,50,52]. While seven studies had serious RoB in different domains. For instance, two studies had serious bias due to confounding factors [37,42], and six studies had serious RoB in the domain of selection of participants [29,33,34,37,40,42,48]. One study had moderate RoB in the domain of selection of participants [44], as described in Figure 8.

Certainty of Evidence

For the prevalence of anxiety among caregivers of Alzheimer’s disease patients based on RCTs and non-RCTs with 630 patients, the certainty of evidence was moderate due to the high heterogeneity. Similarly, for depression (605 patients), the certainty of evidence was moderate due to the high heterogeneity. However, a high level of certainty of evidence was observed in the interventions used for the reduction of neuropsychiatric symptoms among caregivers (Table 5).

**Table 5:** GRADE framework for the assessment of certainty of evidence. RCT: Randomized Controlled Trial, ^a^High heterogeneity, ^b^Substantial heterogeneity.

Certainty assessment							№ of patients		Effect		Certainty	Importance
№ of studies	Study design	Publication bias	Inconsistency	Indirectness	Imprecision	Other considerations			Relative (95% CI)	Absolute (95% CI)		
Anxiety prevalence												
7	RCT and non-RCT	No	Not serious	Not serious	Not serious	None	630		-	MD 0.40 (95% CI, 0.28-0.52	⨁⨁⨁ Moderate^a^	Important
Depression prevalence												
8	RCT and non-RCT	No	Not serious	Not serious	Not serious	None	605		-	MD 0.43 (95% CI, 0.28-0.52	⨁⨁⨁ Moderate^a^	Important
Assessment tools used for impact assessment of different interventions												
8	RCT and non-RCT	No	Not serious	Not serious	Not serious	None	Intervention: 546	Control: 519	-	MD -0.10 (95% CI, -0.96 to 0.76)	⨁⨁⨁⨁ High^b^	Important

Discussion

Caregivers of AD patients are often at risk of developing psychiatric symptoms, such as anxiety, depression, and other behavioral problems, attributed to the multifaceted demands of caregiving and the progressive nature of the disease. Therefore, this meta-analysis aimed to investigate the neuropsychiatric and associated symptoms and management in caregivers of AD patients. Meanwhile, only AD patients were included due to their high global prevalence.

In the present study, psychiatric symptoms, like anxiety and depression, had a significant (p<0.01) impact on the caregivers of AD patients, and the pooled prevalence was 40% and 43%, respectively. Similar findings were observed in another meta-analysis, and the pooled prevalence for depression and anxiety was 34% and 43.6%, respectively [59]. Another study also revealed a positive association of severe psychiatric symptoms in AD patients with stress in the caregivers [60]. A strong (r=0.82, p<0.01) correlation was also observed in a cross-sectional study between the total score on the neuropsychiatric inventory-distress and the total score on the neuropsychiatric inventory, also strong association (r=0.0, p<0.01) between the number of neuropsychiatric symptoms and the total score on the neuropsychiatric inventory distress, which indicated that the patients with higher rate of these symptoms, caregivers distress increases and become more intense [61]. Furthermore, caregiver burden is also considered another predictor for the development of neuropsychiatric symptoms in caregivers and is positively correlated with behavioral disturbance in patients [62]. In contrast, in a cross-sectional study, a non-significant correlation with neuropsychiatric symptoms, like depression and apathy, was observed, and this non-significance may be due to the timely provision of interventions for caregivers [63]. Likewise, when AD patients are treated with anti-AD drugs, these drugs reduce the neuropsychiatric symptoms in patients, and the quality of life of patients becomes better, which ultimately reduces the stress and burden of the caregivers [60]. Meanwhile, it is understood that AD patients with more severe and intense behavioral problems, like memory loss and more dependency, can increase the burden on caregivers, which ultimately leads to the development of neuropsychiatric symptoms. These symptoms become more severe when caregivers feel helpless, experience social isolation, and, most importantly, unpredictable patient behavior like aggression or agitation. Notably, elderly patients with more severe and intense neuropsychiatric symptoms are more associated to experience cognitive impairment and an increase in the severity of the disease, which ultimately reduces the quality of life of the patient and raises the caregiver’s stress level [64]. Furthermore, due to the lack of appropriate and adequate support from family members can result in caregiver burnout and end in the development of depression, anxiety, and sleep disturbance. Moreover, in the present meta-analysis, a non-significant difference (p>0.01) with an effect size of -0.10 was observed between the interventions and control group compared with different assessment tools. However, interventions like education and information were designed, and when caregivers were informed and educated regarding the disease progression and prognosis, their burden levels became reduced than the control group [65]. Similarly, another meta-analysis also demonstrated that the psychosocial management strategies had a moderate pre- and post-effect size in reducing depression [66]. Furthermore, interventions like behavioral activation, cognitive training, and reminiscence were used for reducing the neuropsychiatric symptoms and found a 0.33 effect size pre- and post-test and 0.26 at follow-up by using the GDS assessment tool for depression [67]. Overall, literature provides the effectiveness of interventions in reducing the neuropsychiatric symptoms in caregivers, while in our study, we observed a non-significant difference, which may be due to the variation in the assessment tool used across the studies, and difficult to find a consistent effect. However, the -0.10 effect size is a very small difference, which could suggest that the interventions had minimal impact of interventions or that the controls also had a similar impact because controls were not a placebo or without interventions.

The findings of the present study provide insights into the critical need for targeted management strategies to address the neuropsychiatric symptoms among caregivers of AD patients. As it is highlighted in the present study, caregivers show neuropsychiatric symptoms, like anxiety and depression. Therefore, healthcare providers should implement structured support programs, such as education, counselling, CBT, and support groups, which can foster coping strategies and reduce the risk of anxiety and depression. Furthermore, caregiver-focused initiatives, like financial support, should also be considered to enhance caregivers’ overall well-being and ensure better care for AD patients.

Meanwhile, certain limitations should also be considered before the interpretation of the study. Due to a lack of uniform data, we were unable to perform a meta-analysis to compare different treatment modalities. Another important limitation is the presence of heterogeneity among the studies, which may be due to the study designs, caregivers, and AD patients’ characteristics, sample size, and the relationship of caregivers with patients. Most importantly, variations in the assessment tool used for the assessment of neuropsychiatric symptoms and management strategies. Future studies should focus on overcoming these limitations and also on the identification of more personalized interventions. Longitudinal studies are required to address the progression of neuropsychiatric symptoms. In addition, the use of artificial intelligence for the assessment of these symptoms should also be considered.

## Conclusions

This meta-analysis highlights the importance of the prevalence of neuropsychiatric symptoms, particularly anxiety and depression, among caregivers of AD patients, driven by chronic stress and the burden on caregivers. Different intervention strategies, such as CBT, counselling, and education, have shown promising outcomes. However, when different assessment tools were used for the evaluation of the impact of these interventions, a non-significant difference was observed. These findings underscore the urgent need for multidimensional support programs that address the challenges faced by the caregivers of AD patients.

## Appendices

Appendix 1 

**Table 6:** Literature search from different databases.

PubMed ("neuropsychiatric symptoms"[All Fields] OR "stress"[All Fields] OR "depression"[All Fields] OR "anxiety"[All Fields] OR "sleep disturbance"[All Fields] OR "sleep disorder"[All Fields] OR "obstructive sleep apnea"[All Fields] OR "cognitive impairment"[All Fields] OR "hypertension"[All Fields] OR "hypothyroidism"[All Fields] OR "delusion"[All Fields]) AND ("intervention"[All Fields] OR "management"[All Fields] OR "therapy"[All Fields] OR "anti-psychotic drugs"[All Fields] OR "memantine"[All Fields] OR "omega 3"[All Fields] OR "omega three"[All Fields] OR "vitamin D"[All Fields] OR "vitamin B12"[All Fields] OR "cholinesterase inhibitors"[All Fields] OR "ginkgo biloba"[All Fields]) AND "caregivers"[MeSH Terms] AND "alzheimer disease"[MeSH Terms]
ScienceDirect (“neuropsychiatric symptoms” OR “anxiety” OR “sleep disturbance” OR “cognitive impairment”) AND (“memantine” OR “cholinesterase inhibitors” OR “ginkgo biloba”) AND (“caregivers”) AND (“Alzheimer’s disease”)
Google Scholar (“neuropsychiatric symptoms” OR “stress” OR “depression” OR “anxiety” OR “sleep disturbance” OR “cognitive impairment”) AND (“memantine” OR “omega 3” OR “omega three” OR “vitamin D” OR “vitamin B12” OR “cholinesterase inhibitors” OR “ginkgo biloba”) AND (“caregivers”) AND (“Alzheimer’s disease”)
Scopus (“neuropsychiatric symptoms” OR “stress” OR “depression” OR “anxiety” OR “sleep disturbance” OR “sleep disorder” OR “obstructive sleep apnea” OR “cognitive impairment” OR “hypertension” OR “hypothyroidism” OR “delusion”) AND (“intervention” OR “management” OR “therapy” OR “anti-psychotic drugs” OR “memantine” OR “omega 3” OR “omega three” OR “vitamin D” OR “vitamin B12” OR “cholinesterase inhibitors” OR “ginkgo biloba”) AND (“caregivers” OR “caretakers” OR “nurse” OR “attendant”) AND (“Alzheimer’s disease” OR “AD” OR “Alzheimer’s syndrome” OR “Alzheimer-type dementia” OR “dementia”)
